# THE ROLE OF SOCIAL NETWORKS ON THE RELATION BETWEEN AGE DISCRIMINATION EXPERIENCE AND DEPRESSION: A BUFFER OR A STRESS?

**DOI:** 10.1093/geroni/igae098.3260

**Published:** 2024-12-31

**Authors:** Junmin Park, Yeonjung Lee

**Affiliations:** Chung-Ang University, Seoul, Republic of Korea; Chung-Ang University, Seoul, Republic of Korea

## Abstract

A Confucian culture in Korea, which holds the older people in high esteem has been eroded due to the disruption of the traditional family structure since industrialization and modernization, resulting in the diminished respect for older adults. However, given these cultural background, experiences of age discrimination might be devastating especially for older adults. While social capital theory suggests that individuals obtain material, cultural, and human capital through their social networks, it is unclear whether social networks play a role as a buffer or a stress for the association between age discrimination experience and depression. Therefore, the objective of this study is to examine the moderating role of social networks (formal & informal) in the relationship between age discrimination experience and depression. Specifically, formal social networks are the frequency of participation in civic society, and informal social networks are the frequency of contact and interaction with family and non-family members. Andrew Hayes’ PROCESS macro analytical approach was applied using a selected sample who are aged 65 and older from the 2020 National Survey of Older Koreans (n=10,097). The results show that older adults who experienced age discrimination are likely to have more depressive symptoms. While formal social networks had no significant moderating effect, informal social networks had a significant moderating effect. Specifically, the association between age discrimination experience and depression is stronger at a high frequency of informal social network. Findings have implication that social networks might worsen the consequences of age discrimination for depression among Korean older adults.

